# Insall proximal realignment with/without tibial tubercle osteotomy for recurrent patellar instability yields acceptable medium- to long-term results but risk of osteoarthritis progression is considerable

**DOI:** 10.1186/s40634-022-00502-x

**Published:** 2022-07-06

**Authors:** Per Arne Skarstein Waaler, Truls Jellestad, Trine Hysing-Dahl, Elise Elvehøy, Eivind Inderhaug

**Affiliations:** 1 Department of Orthopaedic Surgery, Førde Health Thrust, Førde Central Hospital, Svanehaugvegen 2, 6812 Førde, Norway; 2 Department of Orthopaedic Surgery, Førde Health Thrust, Lærdal Hospital, Førde, Norway; 3 Department of Rehabilitation, Haraldsplass Deaconess Hospital, Bergen, Norway; 4 Department of Physiotherapy, Førde Health Thrust, Lærdal Hospital, Førde, Norway; 5 Department of Orthopaedic Surgery, Haukeland University Hospital, Bergen, Norway; 6 Department of Clinical Science, Faculty of Medicine, University of Bergen, Bergen, Norway

**Keywords:** Patellar instability, Patellar dislocation, Insall, Proximal realignment, Tibial tubercle osteotomy, Oswestry-Bristol classification, Trochlear dysplasia

## Abstract

**Purpose:**

The purpose of this study was to evaluate clinical and radiological results in patients operated for recurrent patellar instability with a surgical approach consisting of Insall proximal realignment with/without tibial tubercle osteotomy (TTO).

**Methods:**

Patients that underwent surgery for recurrent patellar instability at one centre with a uniform technique between 2004 and 2020 were included. Eligible patients were assessed by clinical examination and the disease-specific Banff Patellofemoral Instability Instrument 2.0 (BPII 2.0). Pre- and postoperative radiographs were analysed for patellofemoral osteoarthritis (OA) according to Iwano. Preoperative Magnetic Resonance Imaging (MRI) and radiographs were analysed for anatomical risk factors for patellar instability. Student t-test, chi-square test and ANOVA-analyses were used to investigate whether anatomical risk factors and/or patient characteristics could predict an inferior outcome.

**Results:**

Forty-six patients (47 knees) were included at a mean follow-up time of 6.6 years (SD 4.6; range 1–17). Mean BPII 2.0 score was 60.4 (SD 18.4; range 26–98), and 10.6% (*n* = 5) had suffered a postoperative redislocation. Progression to evident patellofemoral OA was seen in 15% of the patients (*p* < 0.05). The presence of pathoanatomic risk factors did not correlate with recurrent postoperative instability or inferior BPII 2.0 score at the final evaluation.

**Conclusion:**

Patients treated with the current approach reported acceptable medium- to long-term results, but the risk of patellofemoral OA progression is significant. These findings add to the knowledge of expected outcomes after procedures involving Insall proximal realignment, and can guide clinical decision making for surgeons using similar methods.

**Level of evidence:**

Level IV, case series.

## Introduction

Adolescents and young adults suffering from recurrent patellar instability experience pain and undesired reduction in their level of activity. Current guidelines support that surgery is an important part of the treatment strategy [3]. The Insall proximal realignment procedure has historically been an important soft - tissue technique, and was also proposed for treating quadriceps dysplasia by Dejour et al. [11]. The goal was to strengthen the dynamic stabilisation of the patella by lateralization of the vastus medialis muscle insertion combined with a lateral retinacular release [23]. A systematic review found an acceptable failure rate of 6.3% after such procedures, but indications for surgery, outcome assessment measures, and surgical techniques were highly divergent between studies included in that summary [34].

In light of the focus on the medial patellofemoral ligament (MPFL) as the main ligamentous restraint of lateral excursion of the patella [12], reconstruction of the MPFL (MPFL-R) has largely replaced other soft tissue procedures in current treatment algorithms [3]. Several recent reports also criticise proximal realignment procedures as a non–anatomic approach that might increase the risk for patellofemoral osteoarthritis (OA) [13, 39]. However, few studies have applied validated disease-specific patient reported outcome measures (PROMs) - and radiographic evaluation - to evaluate patients treated with such procedures. The Kujala score, frequently used in evaluating this patient category [26], was originally developed for anterior knee pain. It therefore includes only one question directly inquiring patellar instability. In search of more valid and reliable outcome measures, Banff Patella Instability Instrument 2.0 (BPII 2.0) was recently developed [27].

The aim of the present study is therefore to use BPII 2.0 to evaluate a consecutive series of patients treated with a surgical approach consisting of Insall proximal realignment with/without tibial tubercle osteotomy (TTO). It was hypothesised that excessive pathoanatomic risk factors in patients could predict inferior results and increase the risk of patellofemoral OA progression.

## Materials and methods

All patients surgically treated for recurrent patellar instability at a single centre between 2004 and 2020 were retrospectively identified and included in the study. Exclusion criteria included former stabilising surgery, surgery after first-time patellar dislocations due to osteochondral injuries, patellofemoral pain as indication for surgery, concomitant anterior cruciate ligament reconstruction and procedures involving knee replacement. All surgeries were performed by one out of two experienced knee surgeons. Eligible patients were contacted and offered a follow-up examination with questionnaires, clinical examination, radiographs and functional testing. Ethical approval was obtained from the Regional Committee for Medical and Health Research Ethics West 2020/183361. Written informed consent was obtained from all subjects before enrolment into the study.

### Outcome measures

The incidence of postoperative recurrent patellar instability and reoperations were registered. Questionnaires were administered at the follow-up examination. The BPII 2.0 is a Quality of Life (QOL) score ranging from 0 to 100 where a higher score indicates a higher QOL [27]. The Norwegian version is currently in a validation process, so far demonstrating good psychometric properties in several domains [22]. The Kujala score has also been translated and validated in Norwegian [21]. In addition to the above PROMs, patients graded their overall *satisfaction with the procedure* on a visual analogue scale (VAS) ranging from 0 to 100 [29] where a higher score indicates greater satisfaction.

### Clinical examination and functional testing

Evaluation was performed by two independent examiners not formerly involved in patient care. Body mass index (BMI), varus/valgus alignment of the knee, swelling, crepitus, presence of J-sign and range of motion (ROM) of the hip and knee were assessed. Reverse dynamic patellar apprehension test (ReDPAT) [45] was performed and graded. Return to sport (RTS) readiness assessment included the Lower Quarter-Balance Test (YBT-LQ) [37] and single-legged hop tests [30]. RTS readiness criteria were defined as an anterior reach asymmetry of < 4 cm [37] for the YBT-LQ and leg symmetry index (LSI) > 85% [30] for hop-testing. Patients who had undergone subsequent surgery in the opposite knee were excluded from the RTS analysis.

### Radiological evaluation

Pre- and postoperative radiographs (Merchant view) were evaluated for patellofemoral OA according to the Iwano grading system [2], and evident patellofemoral OA was defined as moderate to very severe joint space narrowing (Iwano grade 2–4) [10]. Patellar morphology was classified according to a simplified Wiberg-Baumgartl classification as grade 1, 2, 3 and “Jägerhut” [8].

Preoperative Magnetic Resonance Imaging (MRI) scans were assessed to identify anatomical risk factors for patellar instability. Lateral Trochlear Inclination (LTI) was evaluated using a modified version of the two-slice technique described by Joseph et al. [25], and values ≤11 degrees were considered as trochlear dysplasia. Using the same cranial image, trochlear dysplasia was evaluated qualitatively according to the Oswestry-Bristol Classification (OBC) [40]. Patellar height was evaluated on MRI by measuring Caton-Deschamps index (CDI) and Patellotrochlear index (PTI) on sagittal images [5], and patella alta was defined as CDI > 1.2 [4] or PTI < 0.18% [1]. The tuberositas tibia-trochlear groove (TT-TG) distance was measured by using a sticky note as described by Camp et al. [6]. The cut - off for an increased TT - TG distance was set to ≥15 mm.

To evaluate interrater reliability, two independent orthopaedic surgeons (P.A.S.W. and E.I.) analysed selected radiological measurements in a blinded fashion. Assessment was then repeated by one of the surgeons after six weeks for calculating the intrarater reliability.

### Surgical technique and postoperative restrictions

The proximal realignment procedure is performed through a 7–8 cm midline incision as previously described by Insall [23], with a 2–3 cm plication of the vastus medialis tendon and medial capsule in a lateral direction. The lateral retinacular release respects the capsule. Before final tightening of the non-absorbable sutures fixating the medial plication, the knee is brought through a full ROM to check patellar tracking and the integrity of the stitches. A concomitant medialization TTO was considered by the surgeon if the patient had a clinically increased q - angle with a subluxated patella throughout the axial MRI series. A procedure involving TTO is done through the same incision extended distally. First the lateral release is performed, followed by an osteotomy of the tibial tubercle, sparing a distal part attached to tendon and fascia. The tubercle is medialized to the point where optimal patellar tracking is achieved (maximum 1.5 cm) and fixed with screws. Finally the medial plication is conducted as described.

Postoperative restrictions allow ROM up to 90 degrees of flexion and brace-free full weight-bearing on a straight knee for the first 4 weeks. Free ROM was then allowed. When an isolated Insall proximal realignment was performed, patients could start full weight-bearing after 4 weeks. When combined with TTO, full weight-bearing was allowed after 6 weeks.

### Statistical analyses

All statistical analyses were performed using the IBM SPSS Statistics for Windows, version 26.0 (IBM Corp). The a priori significance level was set to 0.05. Normality was assessed by the Kolmogorov-Smirnov test. Independent sample t-test was conducted to investigate any relationship between PROMs and (1) the presence/absence of pathoanatomic risk factors (such as trochlear dysplasia, high TT - TG distance and patella alta) and (2) different demographic features. One-way ANOVA analysis was used to explore the impact of categorical pathoanatomic risk factors with three or more groups on PROMs. Pearson *r* for parametric variables or Spearman rho for non-parametric variables were calculated to determine any correlation between PROMs and different pathoanatomic and demographic continuous variables. Chi-square test for independence was used to compare pathoanatomic risk factors in patients with postoperative repeat patellar instability and those who were stable. Inter- and intrarater reliability was calculated and graded by using intraclass correlation coefficient (ICC) for continuous data [28], and Krippendorff’s alpha [17] and percentage of agreement for ordinal data.

## Results

**Fig. 1:**
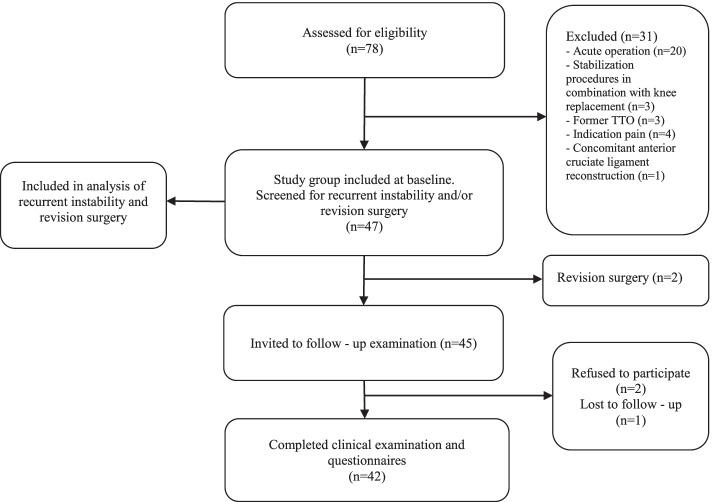
Flow chart for patient selection

Forty-six patients (47 knees) with a mean age of 21.7 years (SD 6.5; range 13–41) were included in the study (Fig. 1), whereof 41 (87.2%) were treated with an isolated Insall procedure. Ninety - 3 % of eligible patients were available for the follow-up examination at a mean of 6.6 years (SD 4.6; range 1–17) after surgery. Additional demographic data are listed in Table 1.

**Table 1:** Demographic patient data (*n* = 42)^a^

	*%*
Female gender	72.3
Right knee treated	44.7
Bilateral instability	40.5
BMI (kg/m2) at surgery, mean (SD; range)	25.2 (4.7; 17–39)
Duration (years) of instability at surgery, mean (SD; range)	7.7 (7.0; 1–28)

^a^Values are presented as (%) unless noted otherwise

### Outcomes at follow-up

At the time of follow-up, 5 patients (10.6%) had suffered a redislocation and further 8 patients (17.0%) described at least one episode with subluxation of the patella. The overall reoperation rate among the included patients was 15.2% (*n* = 7): revision stabilization procedure (*n* = 2), revision of a superficial wound infection (*n* = 1), drainage of a postoperative hematoma (n = 2), hardware removal (n = 1) and knee arthroscopy with removal of scar tissue (n = 2). Mean BPII 2.0 score was 60.4 (SD 18.4; range 26–98), mean Kujala score was 75.4 (SD 14.7; range 40–100) and mean VAS of satisfaction was 78.4 (SD 26.0; range 1–10) at the follow-up evaluation. No significant differences in outcome measurements between surgical procedures (Insall with/without TTO) were found (n.s), see Table 2.

**Table 2:** Between-groups differences (Insall with/without TTO)^a^

	*Insall*	*Insall with TTO*	*P.value*
Pathoanatomical risk factors on MRI	*n = 39*	*n = 5*	
LTI, mean (SD; range)	7.2 (8.4; −14-23)	4.3 (8.6; −14-13)	n.s
TT-TG distance, mean (SD; range)	15.0 (4.8; 7–24)	20.2 (4.9; 16–25)	***p*** **< 0.05**
Caton - Deschamps Index, mean (SD; range)	1.2 (0.2; 0.9–1.7)	1.1 (0.2; 0.9–1.4)	n.s
Outcomes	*n = 41*	*n = 5*	
Postoperative recurrent patellar dislocation (%)	12.2	0	n.s
Reoperations (%)	14.6	20.0	n.s
Patient reported outcome measurements	*n = 37*	*n = 5*	
BPII 2.0 (SD; range)	60.9 (19.0; 26–98)	57.1 (14.9; 45–82)	n.s
Kujala (SD; range)	76.5 (13.4; 52–100)	67.2 (22.0; 40–93)	n.s
VAS of satisfaction (SD; range)	77.3 (26.4; 10–100)	86.0 (23.7; 44–100)	n.s

^a^*SD* Standard deviation, *LTI* Lateral trochlear inclination, *TT-TG* Tuberositas tibia-trochlear groove, *BPII* Banff Patella Instability Instrument, *VAS* Visual analogue scale

### Clinical and radiological findings

Knee ROM deficit ≥10 degrees was more frequently in knees operated with a combined Insall and TTO (*p* < 0.05). Additional clinical findings and RTS readiness rates are summarised in Table 3.

**Table 3:** Clinical findings and return to sport (RTS) readiness at follow-up^a^

	*n*	*%*
Clinical findings	*42*	
Valgus malalignment		40.4
Hyperextension > 10 degrees		9.5
Knee range of motion deficit > 10 degrees		11.9
Increased femoral anteversion^b^		7.1
Positive J - sign		9.7
Positive ReDPAT 0–30 degrees		28.6
Positive ReDPAT > 30 degrees		11.9
Functional tests, return to sports (RTS) readiness	*39*^c^	
Single leg hop test with LSI > 85%		66.7
YBT-LQ anterior reach < 4 cm		79.5
Both RTS criterias accomplished		59.0

^a^*ReDPAT* Reversed dynamic patellar apprehension test, *YBT-LQ* Lower Quarter - balance test, *LSI* Limb symmetry index ^b^ > 70 degrees internal rotation rotation or a difference > 30 between passive internal and external rotation ^c^3 knees were excluded from analysis due to bilateral surgery within a year

Differences in pathoanatomic MRI features between the surgical procedures (Insall with/without TTO) are listed in Table 2. A general high frequency of patellofemoral pathoanatomic risk factors were found on the radiological evaluation (Table 4). A significant progression to evident patellofemoral OA was seen from baseline to the follow-up evaluation - from 9% to 24% respectively (*p* < 0.05).

**Table 4:** Patellofemoral anatomical features on radiographs and MRIs^a^

	*n*^b^	%
Patellas morphology	*44*	
Wiberg 1		0
Wiberg 2		27.3
Wiberg 3		65.9
“Jägerhut”		6.8
Oswestry -Bristol classification of trochlear dysplasia	*44*	
Normal		2.3
Mild		34.1
Moderate		36.4
Severe		27.3
Lateral trochlear inclination angle	*44*	
≤ 11 degrees		65.9
TT-TG distance	*43*	
≥ 15 mm		58.1
Caton - Deschamps Index	*43*	
> 1.2		53.5
Patellotrochlear Index	*43*	
< 18%		2.3

^a^*OBC* Oswestry Bristol Classification, *LTI* Lateral trochlear inclination, *TT-TG* Tuberositas tibia-trochlear groove. ^b^Number of knees evaluated

Substantial intrarater, but low interrater reliability was found for the ordinal variables (OBC and Iwano). Both inter- and intrarater reliability were considered either good or excellent for all the continuous variables (LTI, TT - TG, CDI). Additional information about reliability analyses is provided in Table 5.

**Table 5:** Inter- and intrarater reliability; Krippendorff’s alpha (Kα), %agreement and intraclass correlation coefficient (ICC)^a^

	Interrater reliability			Intrarater reliability		
	***Kα***	*95% CI*	*%agreement*	***Kα***	*95% CI*	*%agreement*
Iwano	**0.62**	0.45–0.78	67.8%	**0.70**	0.55–0.83	73.1%
OBC	**0.65**	0.43–0.83	65.9%	**0.72**	0.60–0.83	61.4%
	***ICC***			***ICC***		
LTI	**0.92**	0.86–0.96	N/A	**0.95**	0.92–0.97	N/A
TT-TG	**0.89**	0.81–0.94	N/A	**0.91**	0.85–0.95	N/A
CDI	**0.72**	0.53–0.84	N/A	**0.80**	0.67–0.89	N/A

^a^*OBC* Oswestry Bristol Classification, *LTI* Lateral trochlear inclination, *TT-TG* Tuberositas tibia - trochlear groove distance, *CDI* Caton–Deschamps index

### Predictors for inferior outcomes

Neither demographic factors, findings at clinical examination nor inability to meet the RTS criteria correlated with an inferior BPII 2.0 (n.s) at follow-up. BPII 2.0 score was slightly higher in patients with follow up ≥3 years compared to those with follow up < 3 years (61.4; SD 16.0; range 26–98 versus 57.9; SD 16.0; range 34.5–92.0), but the difference was not significant (n.s). Further, no differences in scores were found between those with a stable patella and those who had postoperative repeat instability (n.s).

No correlation was found between inferior BPII 2.0 score and (a) severity of trochlear dysplasia, (b) patella alta or (c) increased TT-TG distance. Even if (a)-(c) were combined for analyses, there was no relationship between pathoanatomical features and risk of inferior scores (n.s.). Further, the presence and severity of pathoanatomic factors did not increase the risk of undergoing any later surgery. A higher frequency of postoperative recurrent instability was seen in those with a patella alta (*p* < 0.05) when defined as CDI > 1.2. No such relationship was evident for the other pathoanatomical features. Increasing Wiberg grade of the patella did neither lead to an inferior BPII 2.0 score, nor did it increase the risk of recurrent postoperative instability (n.s).

Four patients had evident preoperative patellofemoral OA. These had a significantly lower BPII 2.0 score at follow-up when compared to the rest of the cohort (42.0 versus 62.1, p < 0.05). There was also a tendency towards lower BPII 2.0 scores in those who had a preoperative Iwano grade 0–1 with a progression to Iwano grade ≥ 2 postoperatively (47.3 versus 62.9, n.s). No significant differences of patellofemoral OA progression were seen in those with long-term (> 5 years) follow-up after surgery compared to those at medium-term (1–5 years) after surgery. Trochlear dysplasia defined as LTI ≤11 degrees did not correlate with progression of patellofemoral OA (n.s).

## Discussion

The main finding of the current study is an overall VAS of satisfaction with surgery of 78.4, a mean BPII 2.0 score of 60.4 and a 10.6% redislocation rate at 6.6 years follow-up after an Insall proximal realignment procedure with/without TTO for patients with recurrent patellar instability. Further, although a non-anatomic surgical approach was applied to the current cohort - pathoanatomical risk factors for instability did not predict inferior clinical outcomes. Overall, the incidence of patellofemoral OA increased substantially from baseline to the mid- to long-term radiologic evaluation.

To date, no other studies have evaluated a treatment algorithm based on Insall proximal realignment with a multitudinous approach, including a validated disease-specific PROM. This allows for comparison across studies applying different surgical approaches for recurrent patellar instability. A few recent studies report BPII 2.0 scores after MPFL-R with/without additional individualised procedures. Both Zimmermann and Börtlein [44] and Hiemstra et al. [18] found an increase in BPII 2.0 score from pre – to postoperatively from 29 and 28, to 76 and 74, respectively. Although the mean postoperative score in these studies is higher compared to the current patient series, the follow-up time is considerably shorter and it is unclear whether patients with preoperative signs of patellofemoral OA were included in those studies. In the current study, patients with evident preoperative OA displayed significantly lower BPII 2.0 scores at the final evaluation, regardless of follow-up time.

Compared to the mean Kujala score of 75.4 in the current study, Efe et al. [14] found a higher mean score of 85 at 4 years in 46 patients treated with Insall proximal realignment. In that study, however, Kujala scores as high as 100 were recorded. This ceiling effect, along with the fact that only one question concerns patellofemoral instability, illustrates the limitations of using the Kujala score as a primary outcome measure when evaluating patients with patellar instability [20].

In the current cohort, 10.6% of patients suffered from a redislocation- with an additional 17.0% describing at least one episode of patellar subluxation throughout the follow-up period. In the comparable study by Efe et al. [14], where patients treated solely with the Insall proximal realignment procedure were included, a higher redislocation rate of 22% was seen. This difference in findings might be due to the inclusion of TTO in the current treatment strategy. In comparison, studies applying modern MPFL-R - regardless of pathoanatomic features of treated knees - redislocation and/or subluxation rates between 3.4% and 8.4% [18, 43] have been seen. One of the few other studies reporting both on luxation and subluxation rates after MPFL-R with/without TTO is that by Enderlein et al. [15]. They observed a redislocation rate of 4.5% and a subluxation rate as high as 39%. Both differences in surgical approach for patellar and heterogeneous definitions of recurrent instability/failure can explain the range in failure rates seen across different patient cohorts [9].

Patients with recurrent patellar instability have an increased risk of developing patellofemoral OA. Even if surgery can reduce the risk of OA development [36], several long-term studies report a significant progression of patellofemoral OA at follow-up evaluation [7, 35, 41]. Recent publications on MPFL-R report minimal patellofemoral OA progression [31, 42]. These, however, often exclude patients with excessive pathoanatomic features or include only short- to medium term results. In the current cohort, progression from Iwano 0–1 to Iwano ≥2 (evident patellofemoral OA) was seen in 15% of the patients. Schuttler et al. [39] reported a somewhat higher rate of patellofemoral OA progression - of 43% - in a patient series treated with an isolated Insall technique. That study also included patients without preoperative radiographs - introducing a potential bias in the reporting. Those with trochlear dysplasia seem to have a particular risk patellofemoral OA development [36], but no such correlation was found in the current patient series.

Detection of one or more common pathoanatomical risk factors on preoperative imaging (trochlear dysplasia, patella alta or increased TT – TG distance) or postoperative clinical examination (valgus malalignment, increased femoral anteversion, positive reDPAT) did not predict an inferior patient reported function in the current study. In fact, the tendency was an inverse relationship displaying better BPII 2.0 scores for those with a more severe trochlear dysplasia. The same tendency was seen when several risk factors were combined. These results correspond to findings by Hiemstra et al. [19], demonstrating how bilateral symptoms and younger age at first dislocation, but not pathoanatomic features, predicted a lower BPII score in patients operated with isolated MPFL-R. Other studies have demonstrated that female gender, higher age and increased BMI at the time of surgery can predict inferior outcomes [9, 15]. Conversely, the current study could not find a significant correlation between any demographic features and inferior BPII 2.0 score.

In the current patient series, patella alta defined as CDI > 1.2 was significantly more present in patients with postoperative recurrence of patellar instability, corroborating results from other surgical approaches [38]. However, only one of these patients had patella alta defined as PTI < 18%, illustrating a potential bias when using measurements developed for lateral radiographs on sagittal MRIs [32]. Other studies have found that trochlear dysplasia, either alone or in combination with other anatomical risk factors such as patella alta, a lateralized tibial tubercle, valgus malalignment and/or increased femoral anteversion, is the most important risk for failure in MPFL-R based treatment algorithms [16]. Yet again, other authors have seen no correlation between recurrent postoperative dislocations and trochlear dysplasia, patella alta and/or increased TT-TG distance [43]**.** These diverging findings might be explained by inherent limitations in methods used to measure the different risk factors [33]. The TT – TG distance, for example, can be influenced by several potential confounding factors, and recently published work suggests that other parameters, such as the tibial tubercle – midepicondyle distance, can be more reliable in predicting recurrent instability after MPFL – reconstruction [24]. Thus, the findings both in the current work and similar studies should be interpreted cautiously.

The current study has several limitations. Most importantly, no PROMs were collected prior to surgery - and there was no control group. Hence, it is not possible to isolate the potential effectiveness of the current surgical approach. Nevertheless, indications for surgery, patient demographic and pathoanatomic features reflect other similar published case series - allowing a cautious extrapolation of treatment effect from the current cohort. Further, patient satisfaction with the procedure indicates an overall effect on their quality of life. Several patients did not have preoperative lateral radiographs available, and CDI had to be measured on MRI. This could lead to a possible bias in the reporting of patella alta, but PTI was also included in the evaluation. MRI scans were obtained from several institutions and differences in protocols can influence measurements. A certain variation, however, reflects everyday life in clinical practice and measurements for evaluating patellar instability should be sturdy enough to overcome such issues. With only a moderate size of this cohort, generalisation of the findings should be done with care. On the other hand, 93% of patients eligible for the study were available at follow-up and these results should therefore reflect well on the population treated for patellar instability at the current clinic.

## Conclusion

This is the first study to report on mid- to long-term results after Insall proximal realignment with/without TTO for recurrent patellar instability applying a multitudinous approach including disease-specific and validated outcome measures. Although one out of three patients experienced some sort of persistent instability and a significant progression of patellofemoral OA was seen, patients treated with the current approach reported an overall good satisfaction with results after surgery and acceptable medium- to long-term results.

## Data Availability

The datasets used and/or analysed during the current study are available from the corresponding author on reasonable request.

## Abbreviations

TTO: Tibial tubercle osteotomy
BPII 2.0: Banff Patellofemoral Instability Instrument 2.0
OA: Osteoarthritis
MRI: Magnetic Resonance Imaging
MPFL: Medial patellofemoral ligament
MPFL-R: Medial patellofemoral ligament reconstruction
PROMs: Patient reported outcome measures
VAS: Visual analogue scale
BMI: Body mass index
ROM: Range of motion
ReDPAT: Reverse dynamic patellar apprehension test
RTS: Return to sport
YBT-LQ: Lower Quarter-Balance Test
QOL: Quality of Life
LTI: Lateral Trochlear Inclination
OBC: Oswestry-Bristol Classification
CDI: Caton-Deschamps index
PTI: Patellotrochlear index
TT-TG: Tuberositas tibia-trochlear groove
ICC: Intraclass correlation coefficient

## References

[1] Ali SA, Helmer R, Terk MR (2009). Patella Alta: lack of correlation between Patellotrochlear cartilage congruence and commonly used patellar height ratios. AJR Am J Roentgenoly.

[2] Allain J, Dejour D (2003). Arthrose fémoro-patellaire isolée. Symposium SOFCOT 2003. Rev Chir Orthop Reparatrice Appar Mot.

[3] Arendt EA, Donell ST, Sillanpää PJ, Feller JA (2017). The management of lateral patellar dislocation: state of the art. J ISAKOS.

[4] Arendt EA, England K, Agel J, Tompkins MA (2017). An analysis of knee anatomic imaging factors associated with primary lateral patellar dislocations. Knee Surg Sports Traumatol Arthrosc.

[5] Biedert RM, Albrecht S (2006). The patellotrochlear index: a new index for assessing patellar height. Knee Surg Sports Traumatol Arthrosc.

[6] Camp CL, Heidenreich MJ, Dahm DL, Bond JR, Collins MS, Krych AJ (2016). A simple method of measuring tibial tubercle to trochlear groove distance on MRI: description of a novel and reliable technique. Knee Surg Sports Traumatol Arthrosc.

[7] Carney JR, Mologne TS, Muldoon M, Cox JS (2005). Long-term evaluation of the roux-Elmslie-Trillat procedure for patellar instability: a 26-year follow-up. Am J Sports Med.

[8] Carson WGJ, James SL, Larson RL, Singer KM, Winternitz WW (1984). Patellofemoral disorders: physical and radiographic evaluation. Part II: radiographic examination. Clin Orthop Relat Res.

[9] Cregar WM, Huddleston HP, Wong SE, Farr J, Yanke AB (2021). Inconsistencies in reporting risk factors for medial patellofemoral ligament reconstruction failure: a systematic review. Am J Sports Med.

[10] deDeugd CM, Pareek A, Krych AJ, Cummings NM, Dahm DL (2017). Outcomes of patellofemoral arthroplasty based on radiographic severity. J Arthroplast.

[11] Dejour H, Walch G, Nove-Josserand L, Guier C (1994). Factors of patellar instability: an anatomic radiographic study. Knee Surg Sports Traumatol Arthrosc.

[12] Desio SM, Burks RT, Bachus KN (1998). Soft tissue restraints to lateral patellar translation in the human knee. Am J Sports Med.

[13] Edmonds EW (2016). Adolescent Patella instability extensor mechanics: Insall extensor realignment versus medial patellofemoral ligament reconstruction. J Pediatr Orthop.

[14] Efe T, Seibold J, Geßlein M, Schüttler K, Schmitt J, Schofer MD, Fuchs-Winkelmann S, Heyse TJ (2012). Non-anatomic proximal realignment for recurrent patellar dislocation does not sufficiently prevent Redislocation. Open Orthop J.

[15] Enderlein D, Nielsen T, Christiansen SE, Faunø P, Lind M (2014). Clinical outcome after reconstruction of the medial patellofemoral ligament in patients with recurrent patella instability. Knee Surg Sports Traumatol Arthrosc.

[16] Feucht MJ, Mehl J, Forkel P, Achtnich A, Schmitt A, Izadpanah K, Imhoff AB, Berthold DP (2020). Failure analysis in patients with patellar Redislocation after primary isolated medial patellofemoral ligament reconstruction. Orthop J Sports Med.

[17] Hayes AF, Krippendorff K (2007). Answering the call for a standard reliability measure for coding data. Commun Methods Meas.

[18] Hiemstra LA, Kerslake S, Lafave MR (2021). Patellar apprehension is reduced in Most but not all patients after successful patellar stabilization. Am J Sports Med.

[19] Hiemstra LA, Kerslake SA, Lafave MR (2019). Influence of risky Pathoanatomy and demographic factors on clinical outcomes after isolated medial patellofemoral ligament reconstruction: a regression analysis. Am J Sports Med.

[20] Hiemstra LA, Page JL, Kerslake S (2019). Patient-reported outcome measures for patellofemoral instability: a critical review. Curr Rev Musculoskelet Med.

[21] Hott A, Liavaag S, Juel NG, Brox JI, Ekeberg OM (2021). The reliability, validity, interpretability, and responsiveness of the Norwegian version of the anterior knee pain scale in patellofemoral pain. Disabil Rehabil.

[22] Hysing-Dahl T, Hysing-Dahl T (2021) Validitet av den norske versjonen av Banff Patella Instability Instrument 2.0. Paper presented at the Annual meeting of the Norwegian Orthopaedic Society (NOF), Oslo, Norway, 27–29 october 2021

[23] Insall JN, Aglietti P, Tria AJ (1983) Patellar pain and incongruence. II: Clinical application. Clin Orthop Relat Res 176:225–232 6851330

[24] Iseki T, Nakayama H, Daimon T, Kambara S, Kanto R, Yamaguchi M, Onishi S, Tachibana T, Yoshiya S (2020). Tibial tubercle–midepicondyle distance can be a better index to predict the outcome of medial patellofemoral ligament reconstruction than tibial tubercle-trochlear groove distance. Arthrosc Sports Med Rehabil.

[25] Joseph SM, Cheng C, Solomito MJ, Pace JL (2020). Lateral trochlear inclination angle: measurement via a 2-image technique to reliably characterize and quantify trochlear dysplasia. Orthop J Sports Med.

[26] Kujala UM, Jaakkola LH, Koskinen SK, Taimela S, Hurme M, Nelimarkka O (1993). Scoring of patellofemoral disorders. Arthroscopy.

[27] Lafave MR, Hiemstra L, Kerslake S (2016). Factor analysis and item reduction of the Banff Patella instability instrument (BPII): introduction of BPII 2.0. Am J Sports Med.

[28] Liljequist D, Elfving B, Skavberg Roaldsen K (2019). Intraclass correlation – a discussion and demonstration of basic features. PLoS One.

[29] Liu JN, Brady JM, Kalbian IL, Strickland SM, Ryan CB, Nguyen JT, Shubin Stein BE (2018). Clinical outcomes after isolated medial patellofemoral ligament reconstruction for patellar instability among patients with trochlear dysplasia. Am J Sports Med.

[30] Ménétrey J, Putman S, Gard S (2014). Return to sport after patellar dislocation or following surgery for patellofemoral instability. Knee Surg Sports Traumatol Arthrosc.

[31] Nomura E, Inoue M, Kobayashi S (2007). Long-term follow-up and knee osteoarthritis change after medial patellofemoral ligament reconstruction for recurrent patellar dislocation. Am J Sports Med.

[32] Pace JL, Cheng C, Joseph SM, Solomito MJ (2020). Effect of trochlear dysplasia on commonly used radiographic parameters to assess patellar instability. Orthop J Sports Med.

[33] Post WR, Fithian DC (2018). Patellofemoral instability: a consensus statement from the AOSSM/PFF patellofemoral instability workshop. Orthop J Sports Med.

[34] Ricchetti ET, Mehta S, Sennett BJ, Huffman GR (2007). Comparison of lateral release versus lateral release with medial soft-tissue realignment for the treatment of recurrent patellar instability: a systematic review. Arthroscopy.

[35] Rouanet T, Gougeon F, Fayard JM, Rémy F, Migaud H, Pasquier G (2015). Sulcus deepening trochleoplasty for patellofemoral instability: a series of 34 cases after 15 years postoperative follow-up. Orthop Traumatol Surg Res.

[36] Sanders TL, Pareek A, Johnson NR, Stuart MJ, Dahm DL, Krych AJ (2017). Patellofemoral arthritis after lateral patellar dislocation: a matched population-based analysis. Am J Sports Med.

[37] Saper MG, Fantozzi P, Bompadre V, Racicot M, Schmale GA (2019). Return-to-sport testing after medial patellofemoral ligament reconstruction in adolescent athletes. Orthop J Sports Med.

[38] Sappey-Marinier E, Sonnery-Cottet B, O’Loughlin P, Ouanezar H, Reina Fernandes L, Kouevidjin B, Thaunat M (2019). Clinical outcomes and predictive factors for failure with isolated MPFL reconstruction for recurrent patellar instability: a series of 211 reconstructions with a minimum follow-up of 3 years. Am J Sports Med.

[39] Schüttler KF, Struewer J, Roessler PP, Gesslein M, Rominger MB, Ziring E, Efe T (2014). Patellofemoral osteoarthritis after Insall’s proximal realignment for recurrent patellar dislocation. Knee Surg Sports Traumatol Arthrosc.

[40] Sharma N, Brown A, Bouras T, Kuiper JH, Eldridge J, Barnett A (2020). The Oswestry-Bristol Classification. Bone Joint J.

[41] Sillanpää PJ, Mattila VM, Visuri T, Mäenpää H, Pihlajamäki H (2011). Patellofemoral osteoarthritis in patients with operative treatment for patellar dislocation: a magnetic resonance-based analysis. Knee Surg Sports Traumatol Arthrosc.

[42] Tscholl PM (2020). Clinical and radiological results after one hundred fifteen MPFL reconstructions with or without tibial tubercle transfer in patients with recurrent patellar dislocation—a mean follow-up of 5.4 years. Int Orthop.

[43] Zhao Z, Wang Y, Li J, Wang H, Bai X, Wang Q, Li Z (2021). Clinical outcomes and prognostic factors in patients with recurrent patellar lateral dislocation treated with isolated medial patellofemoral ligament reconstruction: a retrospective single-center analysis. Orthop J Sports Med.

[44] Zimmermann F, Börtlein J (2020). Patient-reported outcomes after revision surgery for failed medial patellofemoral ligament reconstruction: a matched-pair analysis including correction of predisposing factors. Am J Sports Med.

[45] Zimmermann F, Liebensteiner MC, Balcarek P (2019). The reversed dynamic patellar apprehension test mimics anatomical complexity in lateral patellar instability. Knee Surg Sports Traumatol Arthrosc.

